# Multiple ulcerated submucosal masses in the gastrointestinal tract: a rare presentation of metastatic cutaneous malignant melanoma

**DOI:** 10.1055/a-2268-2354

**Published:** 2024-03-01

**Authors:** Gerly Edson Guzman-Calderon, Luis Marin, Fiorella Monge, Jaime Campos, Jose Rivera, Ronald Mendoza

**Affiliations:** 1 279700 Gastroenterology Unit, Hospital Nacional Edgardo Rebagliati Martins, Lima, Peru; 2 538654 Gastroenterology Unit, Clinica Anglo-Americana, Lima, Peru; 3 279700 Pathology Unit, Hospital Nacional Edgardo Rebagliati Martins, Lima, Peru

Malignant melanoma is the most common metastatic tumor of the gastrointestinal (GI) tract 1 ; it is rarely a primary tumor in the GI tract. Malignant melanoma is more frequently identified in the anus and rectum (31% and 22%, respectively), but it can also be found in the esophagus (6%), stomach (3%), small intestine (2%), and large intestine (1%), as well as in the oronasopharynx (35%) 2 .

We report the case of a 71-year-old woman with a history of nodular malignant melanoma of the right leg, which had been surgically resected 3 years previously. She presented to us with an episode of upper GI bleeding, with melena and hematemesis. Laboratory tests showed a hemoglobin of 6.6 g/dL with a mean cell volume (MCV) of 75 fL, and an albumin of 3.2 mg/dL; liver function tests and coagulation tests were normal. An upper GI endoscopy was performed, and multiple gastric ulcerated submucosal masses were found ( Fig. 1 ), in addition to other nodular lesions on the posterior aspect of the duodenal bulb and in the esophageal introitus ( Fig. 2 ). We decided to perform endoscopic mucosal resection (EMR) of one of the gastric masses ( Fig. 3 ; Video 1 ). A lesion with similar features was later found at the ileocecal valve during a colonoscopy ( Fig. 4 ). Histology showed that all of the lesions were malignant epithelioid neoplasms with atypia; immunohistochemical analyses showed positivity for S100(+) and Melan A(+), which is compatible with metastatic malignant melanoma ( Fig. 5 )

**Fig. 1:**
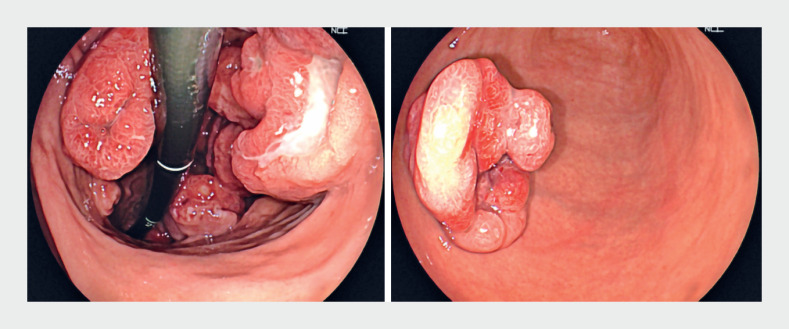
Endoscopic images showing multiple gastric ulcerated submucosal masses.

**Fig. 2:**
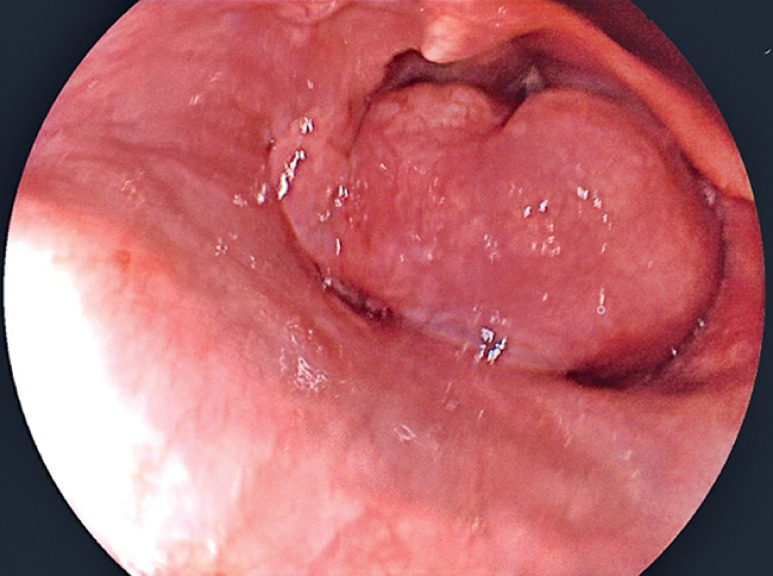
Endoscopic image showing a nodular lesion at the esophageal introitus.

**Fig. 3:**
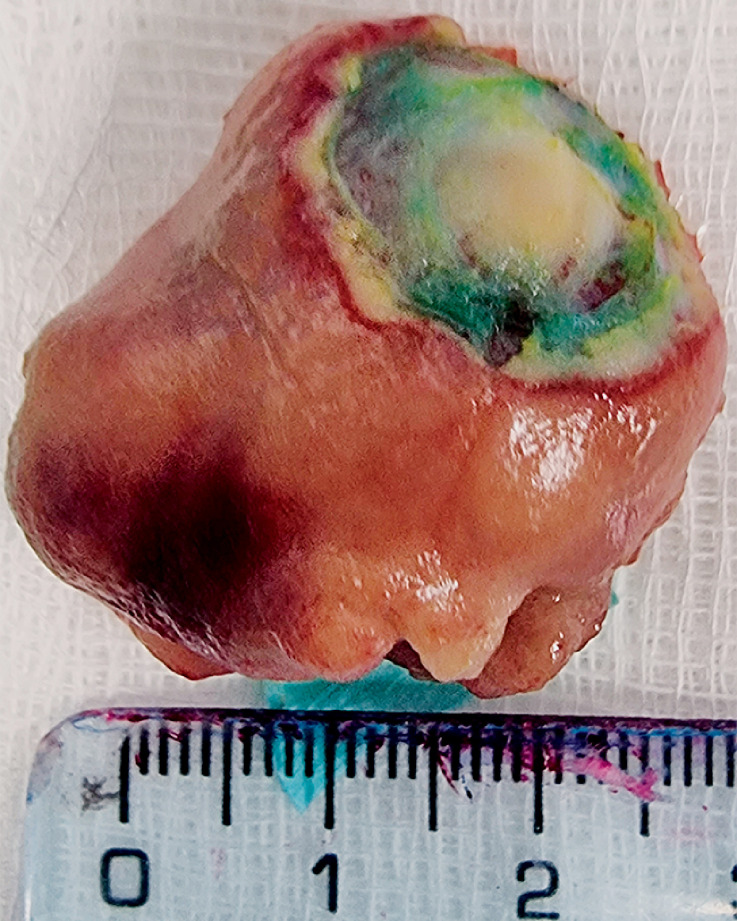
Macroscopic appearance of the gastric nodular mass excised by endoscopic mucosal resection.

Upper gastrointestinal endoscopy is performed showing multiple gastric ulcerated submucosal masses, one of which is excised by endoscopic mucosal resection.Video 1

**Fig. 4:**
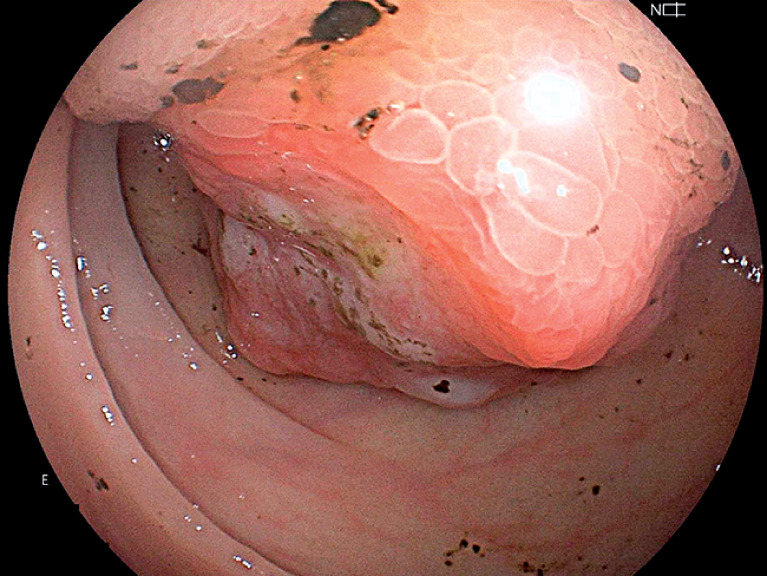
Colonoscopic image showing a lesion with similar features at the ileocecal valve.

**Fig. 5:**
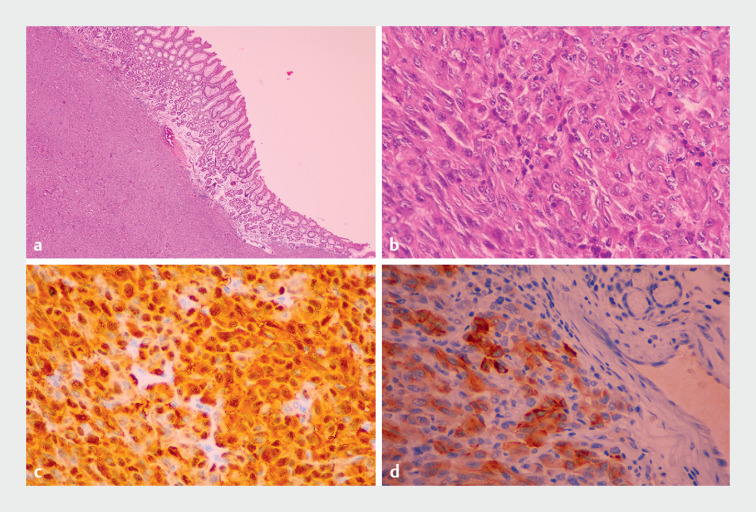
Microscopic appearance of one of the gastric submucosal neoplasms: **a,b** stained with hematoxylin and eosin (H&E) showing: **a** neoplasia involving the gastric submucosa (magnification × 100); **b** tumor cells with extensive eosinophilic cytoplasm, vesicular nucleus, and prominent nucleolus (× 400); **c,d** on immunohistochemical analysis showing positivity of the tumor cells for: **c** S100; **d** Melan A.

Nowadays, it is essential to consider that malignant melanoma is the most common metastatic tumor of the GI tract. Endoscopically, melanoma metastases to the stomach are classified into three types: ulcerated melanotic nodules on normal rugae; ulcerated submucosal masses; and pigmented mass lesions with necrosis 1 3 4 . In this case, the lesions found corresponded with the second type. In conclusion, this case demonstrates a rare presentation of metastatic melanoma, because it was affecting the entire GI tract. It is essential to consider these types of lesions as part of the spectrum of malignant melanoma. They are sometimes asymptomatic but unfortunately the prognosis is quite gloomy if they are found.

Endoscopy_UCTN_Code_TTT_1AO_2AG

## Notes

Endoscopy E-Videos https://eref.thieme.de/e-videos
*E-Videos* is an open access online section of the journal *Endoscopy* , reporting on interesting cases and new techniques in gastroenterological endoscopy. All papers include a high-quality video and are published with a Creative Commons CC-BY license. Endoscopy E-Videos qualify for HINARI discounts and waivers and eligibility is automatically checked during the submission process. We grant 100% waivers to articles whose corresponding authors are based in Group A countries and 50% waivers to those who are based in Group B countries as classified by Research4Life (see: https://www.research4life.org/access/eligibility/ ). This section has its own submission website at https://mc.manuscriptcentral.com/e-videos .

## References

[1] Falk V Zepeda-Gomez S Sultanian R Acute upper gastrointestinal bleeding in a patient with malignant melanoma Case Reports 2018 2018 bcr2018225869 10.1136/bcr-2018-225869 PMC6047700 30002219

[2] Mendes E Costa A Ferreira S The different faces of metastatic melanoma in the gastrointestinal tract Insights Imaging 2022 13 161 36195726 10.1186/s13244-022-01294-5 PMC9532488

[3] Casey S Dvorkin L Alsanjari N Symptomatic malignant melanoma presenting as multiple gastrointestinal polyps BMJ Case Rep 2011 2011 bcr0320102866 10.1136/bcr.03.2010.2866 PMC3062057 22715248

[4] Goral V Ucmak F Yildirim S Malignant melanoma of the stomach presenting in a woman: a case report J Med Case Rep 2011 5 11 10.1186/1752-1947-5-94 21388529 PMC3061929

